# Application of 3-dimensional printing guide template and pointed lotus-style regulator in percutaneous pedicle screw fixation for thoracolumbar fractures

**DOI:** 10.1038/s41598-022-06256-x

**Published:** 2022-02-21

**Authors:** Ming Zhang, Jiayi Li, Tao Fang, Jing Yan, Lungang Wu, Quan Zhou

**Affiliations:** 1 Department of Orthopaedic Surgery, The Affiliated Huai’an Hospital of Xuzhou Medical University, The Second People’s Hospital of Huai’an, NO. 62, Huaihai Road(S.), Huai’an, 223002 China; 2 Department of Orthopaedic Surgery, Nanjing First Hospital, Nanjing Medical University, Nanjing, 210006 China; 3 Department of Orthopaedic Surgery, Changshu First People’s Hospital, Changshu, 215501 China

**Keywords:** Medical research, Trauma, Orthopaedics

## Abstract

This study aims to analysis the efficacy of the 3D printing percutaneous guide template in combination with the pointed lotus-style regulator in percutaneous pedicle screw fixation. 60 thoracolumbar fractures patients receiving percutaneous pedicle screw fixation (PPSF) were enrolled and randomly divided into 3 groups. Patients in Group A received traditional PPSF, while patients in Group B received PPSF with flat end lotus-style regulator and patients in Group C received PPSF with pointed lotus-style regulator. The experimental results showed that the highest number of pedicle screw successfully inserted by the first time was in group C, while lowest in group A (*P* < 0.05). The total time of fluoroscopy and operation were lower in group C, and higher in group A (*P* < 0.05). VAS and ODI scores were all lower after surgery than before surgery in 3groups. VAS and ODI scores were lower in group B and C, compared with group A at day 1, 7 after surgery (*P* < 0.05). KA decreased significantly in 3 groups after surgery and no difference in KA change between 3 groups (*P* > 0.05). Taken together, Application of the 3D printing guide template in combination with pointed lotus-style regulator improved the accuracy of pedicle insertion.

Trial registration: ClinicalTrials.gov Identifier: NCT04980131. Registered 18/07/2021.

## Introduction

Thoracolumbar fractures are most common spine fractures^1,2^. Pedicle screw internal fixation (PSF) is often used for thoracolumbar spinal fractures. With a large incision and extensive injury of paravertebral fascia, muscles and other soft tissues in PSF, patients often suffer low back pain after surgery^3^. With the development of minimally invasive technology, percutaneous pedicle screw fixation (PPSF) has become a more effective surgical method for thoracolumbar compression fractures with short operation time and less soft tissue injury, contributing to a fast recovery^4–7^.

Accuracy of pedicle screw insertion is crucial to PPSF. If placed inaccurately, the pedicle screw may be immobilized to cause injury of the spinal cord, spinal nerves and major blood vessels, resulting in paralysis or even death of the patient^8–10^.

Efforts have been done in improving accuracy of pedicle screw insertion. The computer navigation technology can guide pedicle insertion and pedicle screw placement in real time during operation. But the expensive equipment and the long operation cycle made it difficult in its widespread^11–15^. The mixed reality technology carries out CAD(Computer Aided Design) simulation through the preoperative CT data and provides matched real-time image during the operation^16,17^. However, the system software still needs improving as real-time image is not stable and may not completely fit with the real vertebral body. The application of customized instrument improves the accuracy of positioning, but there still it is not patient-specific^18^. With the development of 3D printing, preoperative CT data can be used for CAD design personalized percutaneous guide template for the positioning operation of pedicle insertion point^19–21^. But low flexity of skin, muscle might cause the misinsertion of the needle^22^.

**Figure 1:**
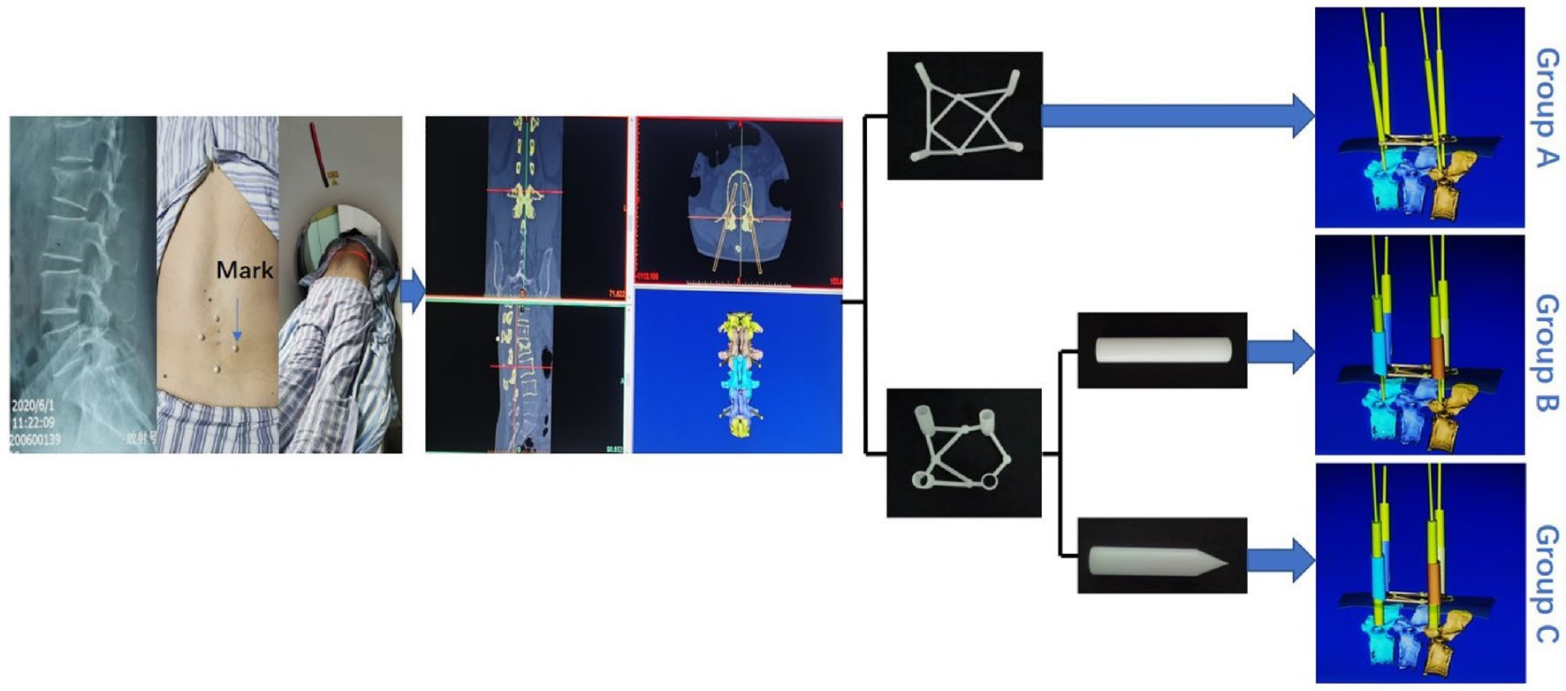
Principles and procedures of percutaneous guide plate and combined with lotus-style regulator (flat ended and pointed) assist puncture positioning.

Previously we designed a flat end lotus-style regulator with porous structure to improve the accuracy and safety of the PPSF^23^. Still, in our study, the rate of pedicle screw successfully inserted by the first time is low, and misinsertion still happened. The template and lotus root regulator can help locate the incision and the angle. However, the location can be affected by the wrong power of the surgeon used for incision, which may cause the failure of first puncture. We hypothesis that the pointed lotus root regulator can locate the puncture point by the pointed tip inserted through the skin to the bone surface of the affected vertebral body, thus improve the accuracy of the puncture (Fig. 1).

## Methods

### Patients

60 patients (27 males, 33 females; average age 55.00 ± 2.22 years old) who underwent PPSF in Xuzhou medical university affiliated Huai 'an hospital were enrolled from June 2018 to June 2020. The informed consents were all signed. This trial was approved by the ethics committee of Xuzhou medical university affiliated Huai'an hospital (HEYLL201705).

This study was a prospective study and randomized by random sequence method. 60 patients were divided into 3 groups, 20 patients (9 male, 11 female; average age 53.00 ± 2.51 years old) in group A received traditional PPSF (affected segment: T_11_ 1, T_12_ 3, L_1_ 8, L_2_ 8); 20 patients (10 male, 10 female; average age 51.63 ± 2.72 years old) in group B received PPSF with flat end lotus-style regulator (affected segment: T_11_ 1, T_12_ 2, L_1_ 11, L_2_ 6); 20 patients (12 male, 8 female; average age 52.00 ± 2.93 years old) in group C received PPSF with pointed lotus-style regulator (affected segment: T_11_ 2, T_12_ 3, L_1_ 9, L_2_ 6).

### Lotus root regulator and guide template preparation

**Figure 2:**
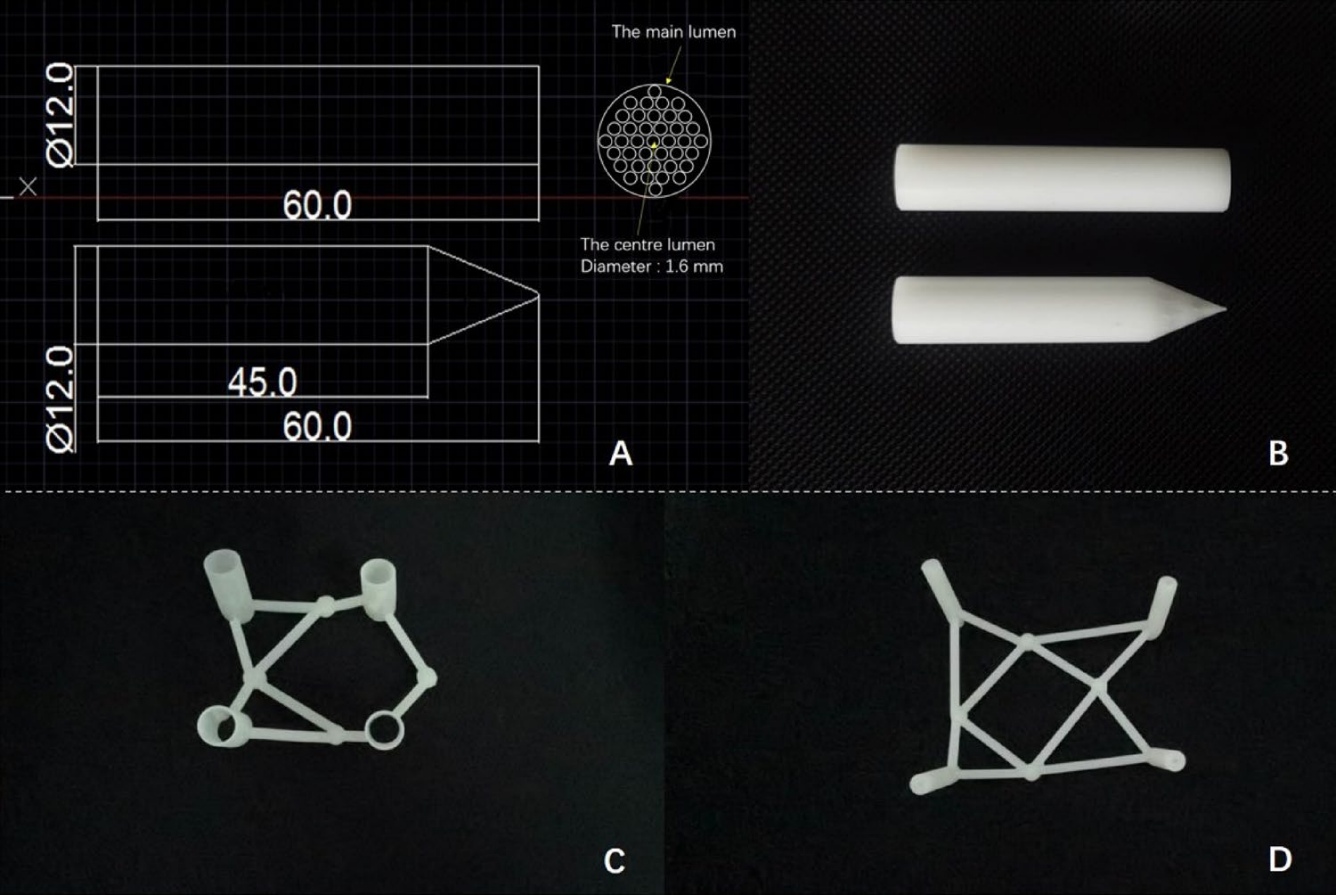
(**A**) Design of pointed lots-style regulator. (**B**) side view of the lotus-type regulators. (**C**, **D**) 3D printed percutaneous guide plates.

Pointed lotus-style regulator was designed and manufactured by Anbang COMPANY (Fig. 2A, B). The 3D printing guide template was designed with CT data of each corresponding patient by MIMICS19.0 and Magics21.0 software (Materialise, Belgium) and printed by 3D printer (Shanghai pulisheng electromechanical technology co., LTD., China) in the key laboratory of medical 3D printing in huai 'an city (Fig. 2C, D). The parameters of the pointed lotus-style regulator and the guide template was listed in Table 1.

**Table 1:** Characteristics of the porous polyoxymethylene thermoplastic regulator and the percutaneous guide template.

Material	Flat end lotus-style regulator	Pointed lotus-style regulator	Combined Percutaneous guide template	Independent Percutaneous guide template
Material composition	Polyoxymethylene	Polyoxymethylene	Photosensitive resin	Photosensitive resin
Material parameter	Diameter of the main lumen: 12 mm; Diameter of the sub-lumen: 1.6 mm; Length: 60 mm	Diameter of the main lumen: 12 mm; Diameter of the sub-lumen: 1.6 mm; Length: 60 mm; Cutting edge length: 15 mm	Outer diameter: 15 mm; Inner diameter: 13 mm	Outer diameter: 3 mm; Inner diameter: 2 mm
Physicochemical properties	Solid, hard texture	Solid, hard texture	Solid, hard texture	Solid, hard texture
Physicochemical properties	No potential cytotoxicity or allergic reaction	No potential cytotoxicity or allergic reaction	No potential cytotoxicity or allergic reaction	No potential cytotoxicity or allergic reaction

### Surgical procedures

#### Preoperative preparation

Baseline data such as gender, age, weight, degree of vertebral body compression, KA, preoperative VAS and ODI scores, and position level of the affected vertebrae was collected before surgery. All patients were operated by one experienced surgeon.

#### Pointed lotus root regulator and guide template manufacture

**Figure 3:**
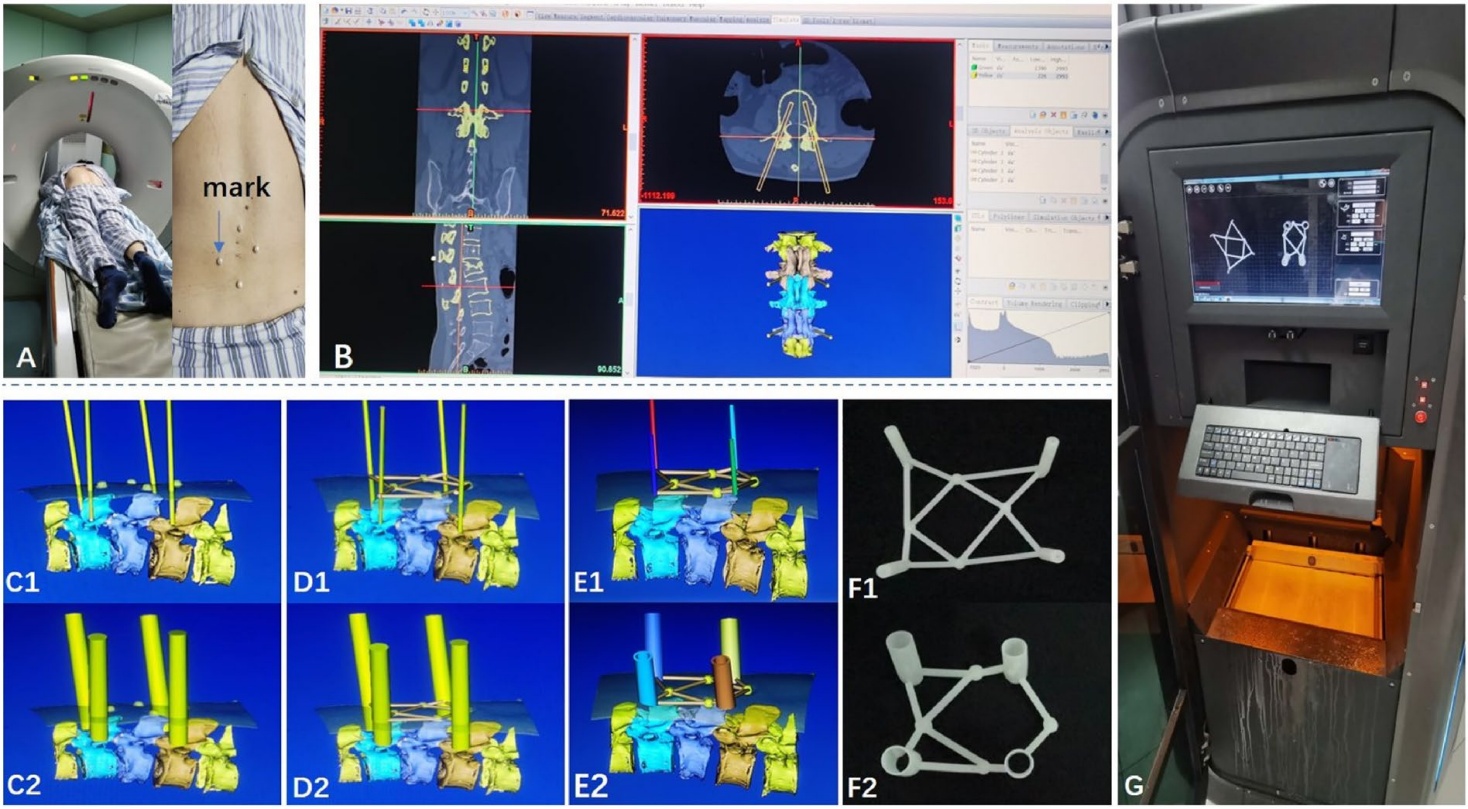
The main process of percutaneous guide plate design and 3D printing. (**A**) Preoperative localization of markers was performed by CT scan. (**B**–**E**) CAD of the main process of personalized percutaneous guide plate. (**F**, **G**) 3D printer is printing the real percutaneous guide plate.

The area of the fracture vertebral body marked by four hemispherical pearls (diameter of 1 cm) at four points 4 cm away from the center of it (Fig. 3A) and scanned by CT. The DICOM file was reconstructed by MIMICS19.0. Insertion point and insertion angle was accurately select to make sure that the inner column was in vertebral body in coronal plane, sagittal plane and transverse plane (Fig. 3B). The inner diameter of column was 2 mm in traditional PPSF(Fig. 3C1) and was increased to 13 cm as required in template guided PPSF(Fig. 3C2). Four hollow navigation tubes were obtained at four marking points (Fig. 3D). The percutaneous guide template was designed by Magics21.0 software (Fig. 3E), and printed by 3D printer (Fig. 3G) with photosensitive resin material (Fig. 3F).

#### Guide template aided PPSF (group A)

**Figure 4:**
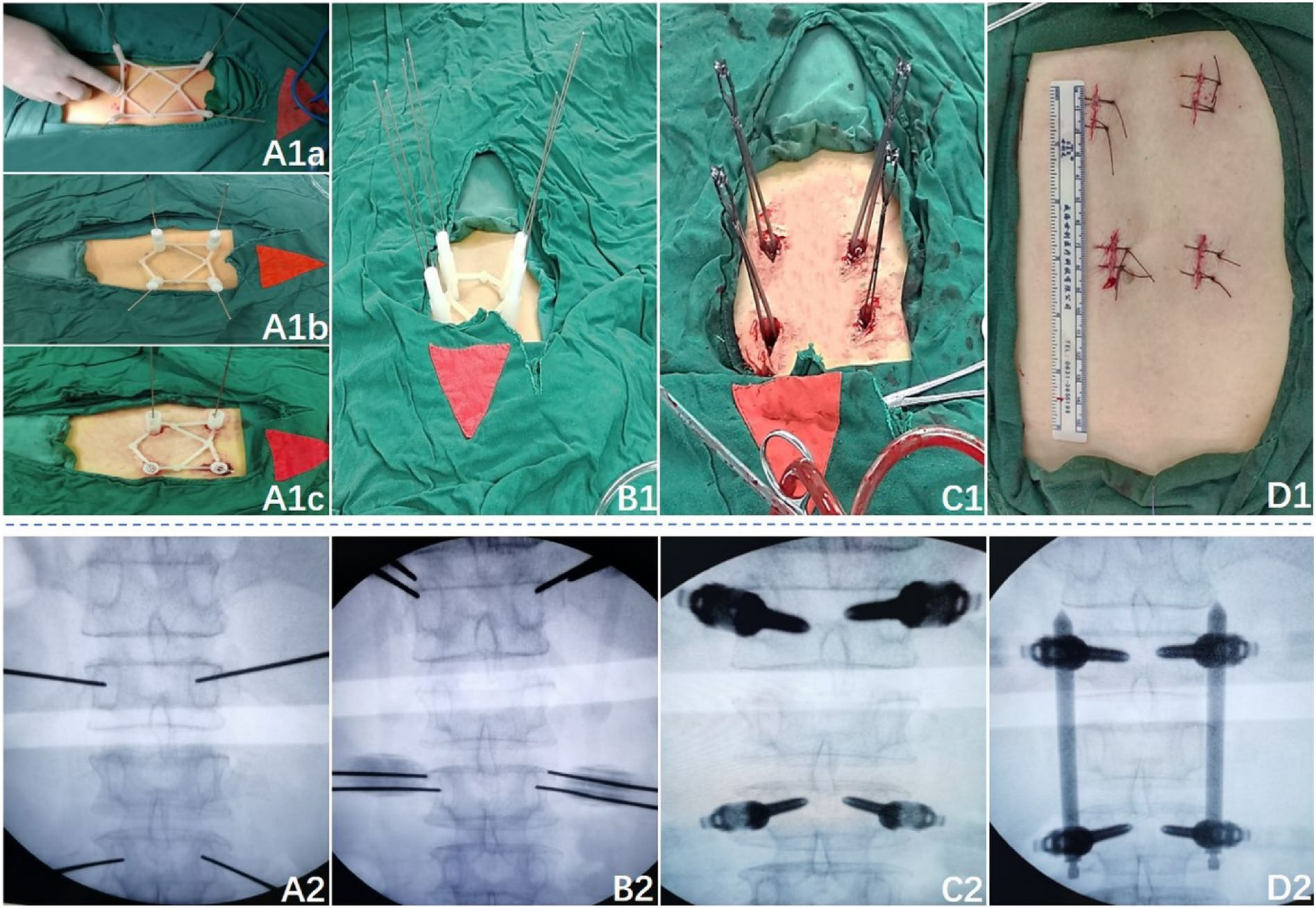
(**A1**–**D1**) The main steps of percutaneous guide combined with pointed lotus-style regulator in PPSF. (**A2**–**D2**) The perspective image of the corresponding step.

After anaesthesia, patients were placed in prone position. To obtain partial reduction and increase lordosis, pads were placed beneath chest and pelvis. The target vertebra was determined by c-arm fluoroscopy, and the needle was inserted according to the marked points (Fig. 4A1a), insertion spot and angle were observed through c-arm perspective (Fig. 4A2), continuing insertion until the needle reaches the front one third of the vertebral body when the angle was satisfactory and keep adjusting when it was not. Cancellous pedicle screws were then inserted (Fig. 4C1,C2). And finally, the prebent connecting rods was installed (Fig. 4D1,D2).

#### Flat ended lotus root regulator and guide template aided PPSF (group B)

The percutaneous guide template was placed as group A and then the four lotus root regulators were fit into the four navigation tubes. Four insertion needles with a diameter of 1.5 mm were inserted into the surface of the bone cortex along the central tube hole of the lotus root regulator, and then slightly into the bone cortex (Fig. 4A1b). C-arm fluoroscopy was used to observe the position and Angle of the insertion needles (Fig. 4A2). Adjust the insert angle by flat ended lotus root regulator without moving the template when the angle was unsatisfactory (Fig. 4B1,B2).The rest procedure was the same as group A.

#### Pointed lotus root regulator and guide template aided PPSF (group C)

The template was placed as Group A and the skin underneath the navigation tube were incised and pointed lotus root regulator were inserted through the navigation tube and through the skin to reach the vertebra and slightly insert into cancellous (Fig. 4A1c), C-arm fluoroscopy was used to observe the position and Angle of the insertion needles (Fig. 4A2). The insertion angle was adjusted with flat ended lotus root regulator without moving the template when the angle was unsatisfactory (Fig. 4B1,B2).The rest procedure was the same as group A.

### Main observational parameters

**Figure 5:**
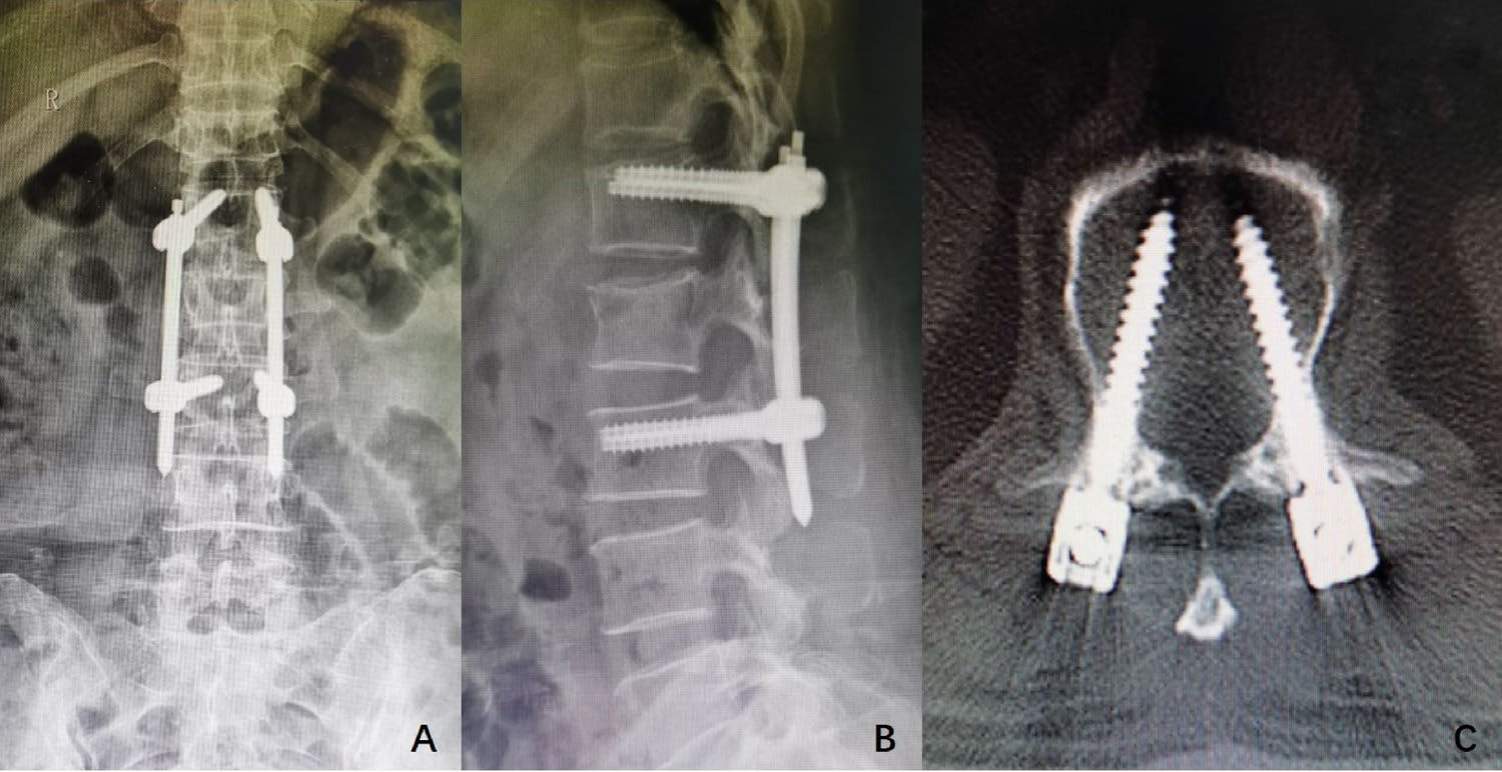
(**A**, **B**) X-ray of group C 3 days after operation, and the height of the affected vertebra recovered well after operation. (**C**) CT scan of group C 3 days after operation, and the pedicle screw is in the padicle.

X-ray and CT scan were taken to observe the pedicle position and the height recovery of the affected vertebral body (Fig. 5A,B,C). The number of successful pedicle instrumented, insertion times before the insertion needle reached the ideal position, total fluoroscopy times, and total intraoperative bleeding were recorded. VAS and ODI scores at 1 day before surgery, 1 day after surgery, 7 days after surgery, 1 month and 3 months and 6 months after surgery; Vertebral pedicle insertion number and KA change rate were also recorded.

### Statistical analysis

SPASS19.0 software was used for statistical analysis, and measurement data were present as X ± S. Paired design *t*-test was used for comparison of measurement data within the group, two independent samples *t*-test was used for comparison of measurement data between the two groups, χ2 test was used for comparison of enumeration data, and rank sum test was used for comparison of qualitative data. *p*-value < 0.05 was considered with statistical difference.

### Statements

All persons were informed in which the article contained information or images that may lead to the identification of the study participants and everyone consented to the relevant identification information/images published in the online open publication.

We confirm that all methods were carried out in accordance with relevant guidelines and regulations.

## Results

### Baseline data

60 patients were randomly divided into 3 groups (male/female = 27/33), 20 patients in each group. There were no statistically significant differences between the 3 groups in gender, age, weight, degree of vertebral body compression, KA, preoperative VAS and ODI scores, position level of the affected vertebrae and width of pedicle (*P* > 0.05)(Table 2).

**Table 2:** Demographic and clinicopathological data of group A, group B and group C.

Patient characteristics	Group A	Group B	Group C	Statistic	*p*-value
Age(yrs)	53.0 ± 2.5	51.6 ± 2.7	52.0 ± 2.9	F = 0.545	0.588
**Gender(n)**				χ^2^ = 0.623^a^	0.733
Male	9	10	12		
Female	11	10	8		
Weight(kg)	69.0 ± 5.1	69.1 ± 6.5	71.4 ± 4.5	F = 0.364	0.699
Degree of vertebral compression(%)	43.8 ± 7.5	43.9 ± 5.8	44.4 ± 6.0	F = 0.023	0.978
Preoperative KA(°)	26.8 ± 4.4	28.3 ± 3.4	29.7 ± 3.1	F = 1.275	0.300
Preoperative VAS scores	7.9 ± 1.3	7.6 ± 1.1	8.0 ± 1.5	F = 0.176	0.840
Preoperative ODI scores	87.5 ± 1.8	88.8 ± 2.1	87.3 ± 1.5	F = 1.572	0.231
**Level of injured vertebra(n)**				χ^2^ = 3.247	0.197
T11	1	1	2		
T12	3	2	3		
L1	8	11	9		
L2	8	6	6		
**Width of pedicle (mm)**					
T11	5.6 ± 1.4	5.8 ± 1.8	5.5 ± 1.2	F = 1.038	0.575
T12	6.4 ± 1.6	6.1 ± 1.1	6.3 ± 1.3	F = 1.990	0.313
L1	4.6 ± 0.6	4.6 ± 0.8	4.8 ± 0.7	F = 0.667	0.745
L2	5.4 ± 0.7	5.5 ± 0.7	5.3 ± 1.1	F = 0.062	0.941
**AO spine classification (n)**				χ^2^ = 1.558^a^	0.459
A1.2	15	16	18		
A1.3	5	4	2		

### Intraoperative parameters

All the operations were successfully completed. The number of pedicles successfully first pierced in group C was higher than that in group A and group B (*P* < 0.05); The number of insertion, fluoroscopy and operation time before the insertion needle reached the ideal position, the total number and time of intraoperative fluoroscopy, and the intraoperative blood loss in group C were all significantly lower than those of group A and group B (*P* < 0.05) (Table 3).

**Table 3:** Comparison of the intraoperative parameters in group A, group B and group C.

Intraoperative clinical indicators	Group A	Group B	Group C	Statistic	*p*-value
First puncture success rate	13/80	(15/80)^b^	(51/80)^ab^	χ^2^ = 51.778^a^	0.00
Number of insertions before reaching the desired position (n)	15.5 ± 2.1	(9.8 ± 1.2)^ab^	(6.2 ± 1.0)^ab^	F = 60.00	0.00
Number of fluoroscopy before reaching the desired position(n)	8.5 ± 0.8	(5.8 ± 0.4)^ab^	(3.2 ± 0.4)^ab^	F = 123.86	0.00
Radiation dosage before reaching the desired position (mSv)	0.9 ± 0.1	(0.6 ± 0.1)^ab^	(0.3 ± 0.1)^ab^	F = 123.87	0.00
Operation time before reaching the desired position (min)	21.0 ± 2.2	(15.2 ± 2.6)^ab^	(8.7 ± 2.1)^ab^	F = 42.73	0.00
Total number of fluoroscopy(n)	16.8 ± 1.0	(13.7 ± 1.0)^ab^	(11.2 ± 1.2)^ab^	F = 42.70	0.00
Total radiation dosage of fluoroscopy (mSv)	1.7 ± 0.1	(1.4 ± 0.1)^ab^	(1.1 ± 0.1)^ab^	F = 42.69	0.00
Total operation time (min)	72.7 ± 2.5	(67.2 ± 2.6)^ab^	(60.7 ± 2.0)^ab^	F = 37.11	0.00
Intraoperative blood loss (ml)	78.2 ± 4.2	76.7 ± 3.7	80.2 ± 5.3	F = 0.94	0.41

Group A is the control group; ^a^Adjusted *P* < 0.05 different from the group A; ^b^Adjusted *P* < 0.05 different from the other groups.

### Postoperative parameters

**Figure 6:**
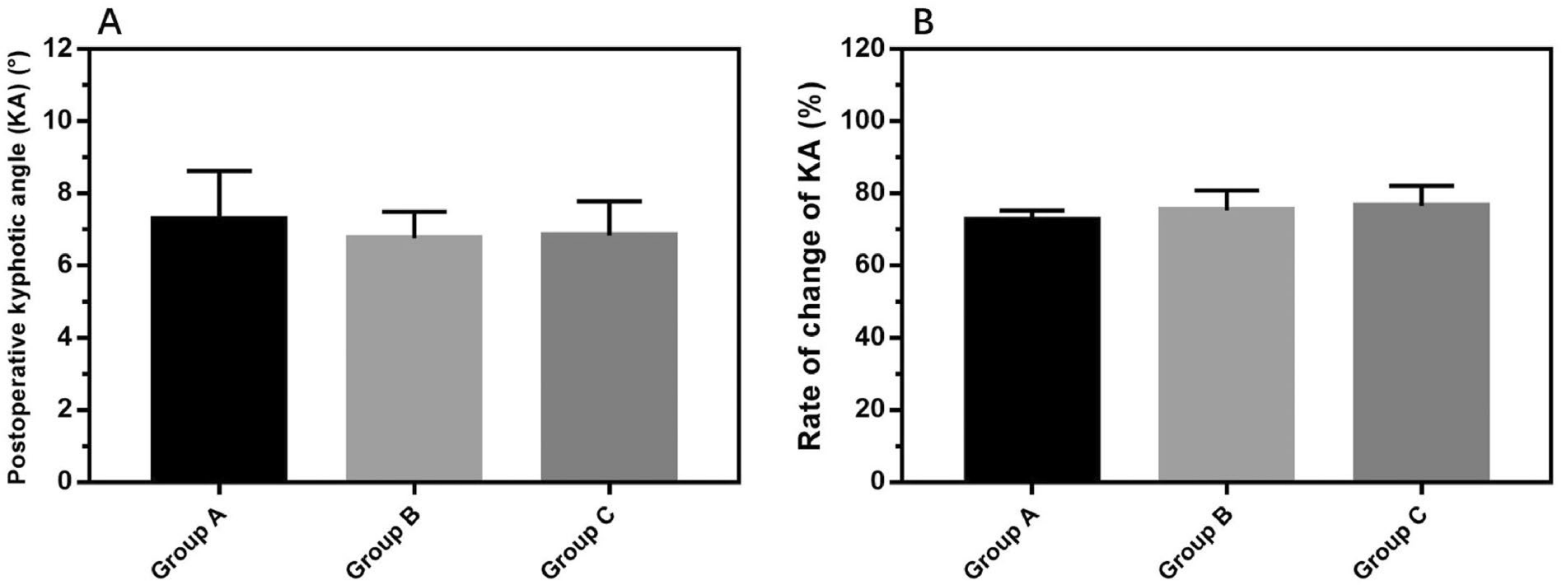
(**A**) The postoperative kyphotic angle(KA) of three groups. (**B**) Rate of change of KA among group A, group B and group C.

**Figure 7:**
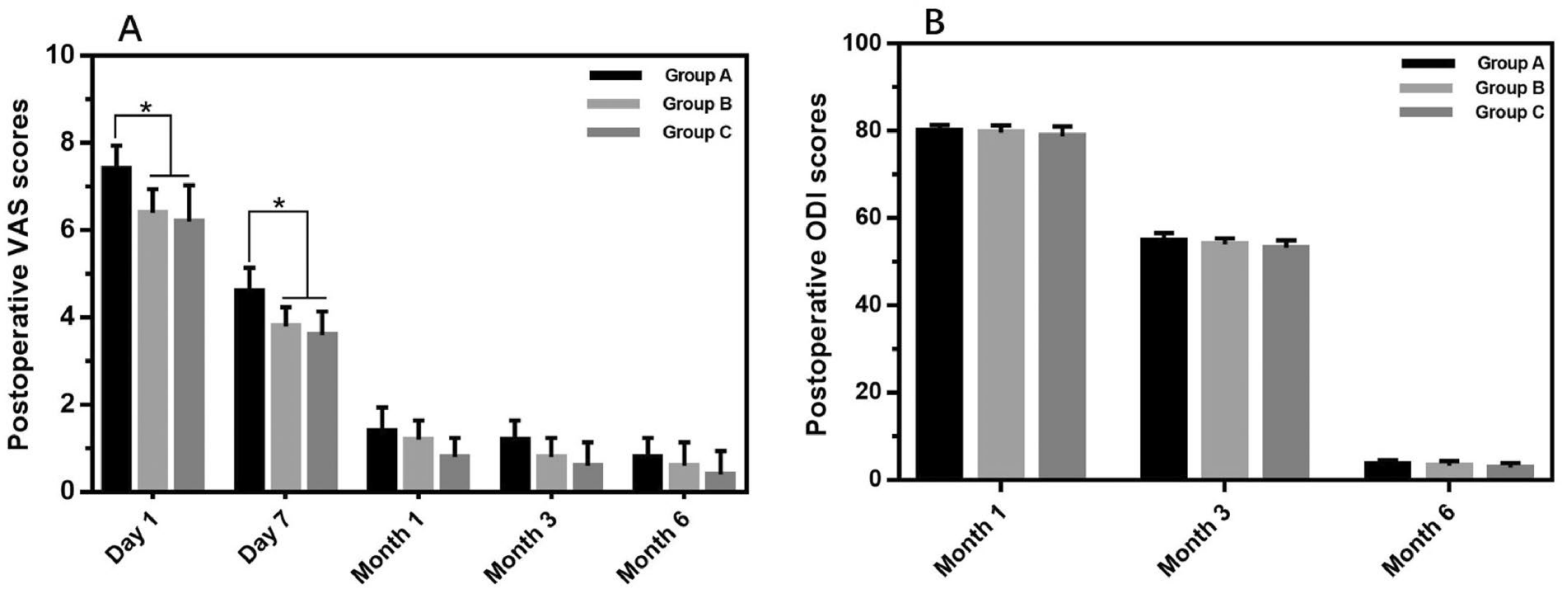
(**A**) Postoperative VAS scores in the three groups in 1 day, 7 day, 1 month, 3 month and 6 month after surgery. (**B**) Postoperative ODI scores in the three groups in 1 month, 3 month and 6 month after surgery. All data represent the mean ± standard deviation. (0.001 < *p < 0.05, 0.001 < **p < 0.01, and ***p < 0.001).

No patients in 3 groups developed neurologic damage. X ray results revealed that the height recovery of the affected vertebral body was obvious in all groups, and there was no difference in KA and KA change rate in all groups (*P* > 0.05) (Fig. 6). All patients were followed up 6 months after surgery. VAS and ODI scores at 1 day and 7 days after surgery were higher in Group A than those in other groups (*P* < 0.05), but no differences were found VAS and ODI scores at 1, 3 and 6 months after surgery in 3 groups (Fig. 7).

## Discussion

PPSF is widely used in treating mild and severe spinal compression fractures, as it can recover the spinal height and achieve immediate spinal stability^24,25^.

With the development of minimally invasive concepts and medical instruments, percutaneous pedicle surgery provides a new idea for the treatment of vertebral compression fractures. Compared with traditional pedicle screw fixation, percutaneous pedicle surgery has the advantages of less trauma and faster postoperative recovery^26–28^, For the whole PPSF operation, pedicle positioning and puncture is extremely important. Accurate positioning and puncture can correctly guide the placement of percutaneous pedicle screw. Otherwise, fixation failure may result, or even damage the spinal cord, nerves and other important tissues^29^. Furthermore, accurate position and puncture of the pedicle has become more and more important.

Fady J. Aky et al.^30^ successfully inserted pedicle screws into 105 patients using computer navigation. The trial showed that the operation time was significantly shorter in the experimental group than in the control group, and the rate of pedicle screw dislocation was significantly lower than in the control group (1.0% vs. 3.3%), demonstrating the accuracy of computer navigation in pedicle puncture. Jing-ye Wu et al.^31^ successfully inserted 24 pedicle screws into 10 patients by using robot-assisted technology. the test results showed that the intraoperative navigation precision translation error 1.09 ± 0.17 mm, Angle error2.17° ± 0.39°, no postoperative neurological complications, proved that the robot assisted technology in the accuracy and safety in the process of pedicle puncture. Although the accuracy of pedicle puncture is improved and the operation risk is reduced to by robot and computer navigation technology, it also has its limitations compared with 3-Dimensional printing guide template and pointed lotus-style regulator in this study. First of all, the cost of machine and equipment is high. Ordinary small and medium-sized hospitals have no economic ability to purchase this equipment, and the charge of intraoperative equipment is high, which increases the medical economic burden of patients to a certain extent and is not conducive to the wide promotion of technical equipment. However, the cost of the combined device we designed and studied is only 200–300 yuan, and there is no use fee of equipment. This is greatly conducive to the popularization and application of the technology and equipment. Secondly, robot and computer navigation technology have high requirements for operators. They not only need strong clinical operation experience, but also need a long time of training and learning to master the technology and carry out operation practice. The learning curve is long, which is not friendly to most clinicians. The surgical auxiliary equipment in this study is simple to operate and has a short learning curve, which can be operated by doctors with certain surgical experience. Third, robot and computer navigation technology need to carry out multiple fluoroscopy during the operation to maintain real-time image update, so as to facilitate the accurate puncture of pedicle, but this will bring relatively more radiation both to doctors and patients. In this study, navigated puncture can be carried out through the patient's CT data before operation, and the percutaneous guide plate matching the position and angle with the patient's puncture can be printed, and a small range of adjustment can be carried out during operation through the tip lotus regulator, so as to achieve the accurate puncture of pedicle, which reduces the amount of radiation during operation.

Liming Wang, et al.^16^ applied the mixed reality (MR) technology to PKP surgery for osteoporotic thoracolumbar compression fractures. Compared with the conventional surgery group, the experimental group could significantly improve the accuracy of pedicle puncture, and effectively reduce the numbers of puncture, fluoroscopy and operation time. However, real-time image signal instability and even image loss exist in MR system during operation, so the stability of the system needs to be further optimized. A. Prokop et al.^18^ treated 36 patients with thoracolumbar compression fractures by the minimally invasive treatment, compared with the control group, experimental group integrated by percutaneous pedicle screws were accurate placement corresponding to the thoracolumbar vertebral body, the average operation time were 49 min and the blood loss was 10–20 ml, the test showed the custom instrument in shorten the operation time, reduce the bleeding and other advantages, but due to the patient and each segment of the vertebral body specificity, custom equipment in use process will appear different degree of error with a custom instrument sextant. Qi Fei, et al.^22^ using 3D printing, computer simulations based on patients with preoperative CT scan data puncture, and print out the individualized percutaneous plate used for auxiliary pedicle puncture, the results shown, percutaneous guide assisted surgery effectively reduces the number of intraoperative fluoroscopy and radiation, shorten the operation time, is beneficial to patients with postoperative recovery, this proved that percutaneous guide in order to increase the effectiveness of pedicle puncture accuracy. But because the soft tissues such as skin, muscle, portability and scalability, in the process of percutaneous guide assisted pedicle puncture, the needle will often appear deviation, make the first wear success rate is not ideal, and as a result of percutaneous navigation guide tube single pipe design, not easy to adjust puncture, later still need to use the C arm machine multiple perspective in order to achieve the ideal puncture point and Angle positioning.

Aiming at the puncture error and the low success rate of the first puncture caused by the skin, muscle and other soft tissue to the percutaneous guide plate and the flat end lotus-style regulator. In this experiment, our research group optimized the design of the lotus-style regulator, the regulator sophisticated processing and combined with percutaneous plate at one end, and trial by percutaneous puncture positioning plate, then insert the porous regulator cutting-edge and reach to puncture the pedicle bone, and to verify its accuracy and the pedicle core-needle security.

Our results showed that the first accurate insertion rate, puncture times to reach the ideal position, number of perspective, radiation dose, operation time and total number of perspective, total dose radiation and surgery total time, etc., were better in group C than in group A and group B, indicating that percutaneous plate combined with pointed lotus root regulator can effectively improve the accuracy of pedicle puncture and then effectively shortened the pedicle puncture times, number of perspective, operation time, reduce the radiation dose, this was because the type of lotus root tip type regulator under the guidance of percutaneous navigation guide tube reach pedicle bone surface, To the greatest extent, the interference of skin, muscle and other soft tissue to the needle can be avoided, so as to achieve accurate pedicle puncture. The insertion times was lower in group B, C than A, which implies that the pointed lotus root regulator can effectively improve the accuracy of the puncture. When the first insertion fails, group A can only move the whole template to adjust. When group B, C only need move the lotus root regulator by guidance of fluoroscope with the whole template keep stay. The insertion accuracy improves by the times because the first insertion, accurate or not, provide a relatively accurate location of the puncture. The insertion after first time only need minor adjust. On the one hand, percutaneous guide template combined with lotus root regulator can help surgeons improve the accuracy of puncture during the operation. However, due to the errors caused by the movement of soft tissues such as skin and muscle, adjusting the puncture still needed to be performed by experienced surgeon. On the other hand, after the completion of the pedicle puncture of the responsible vertebral body, the other steps of PPSF were more likely to be performed by experienced surgeons to shorten the operation time and improve the safety of the operation.

Test results, in terms of intraoperative blood loss, no difference between the three groups, which suggests that the early stage of the sophisticated type lotus root type regulator insert will not bring to patients with massive hemorrhage, this may be due to sophisticated type lotus root type regulator in small incision, to a certain extent of vascular tissue caused by oppression, so there would not be a lot of bleeding. During the operation, the application of percutaneous guide template combined with lotus root regulator can improve the accuracy of pedicle puncture, reduce the total number of intraoperative puncture, resulting in a corresponding reduction in the total number of fluoroscopy, thus reducing the total dose of radiation to surgeons and patients.

Our results showed that there was no significant difference in postoperative KA and KA change rate among the three groups, indicating that PPSF under the three auxiliary methods could effectively fix the diseased vertebra and restore the height of the diseased vertebra, showing the same advantage. B and C group in 1 day, 7 days of VAS score higher than group A and no obvious difference between group B and C, B and C group of short-term on lower back pain relief after surgery was better than that of group A, this may be due to puncture the number of B and C group was obviously less than group A, for soft tissue damage and vertebrae lighter, postoperative lumbar back pain from group A was obvious. The results showed that there was no significant difference in VAS and ODI scores of the three groups at 1 month, 3 months and 6 months after surgery, indicating that there was no significant difference in long-term relief of lower back pain after surgery among the three groups. This might be due to the fact that soft tissue and bone could be effectively recovered after a long time, and lower back pain could be effectively relieved compared with before. Postoperative patients in all three groups showed no obvious symptoms of nerve injury, indicating that percutaneous pedicle screw could be placed smoothly and safely along the puncture needle with the assistance of the three devices, and the three groups showed the same safety.

We also recognize the limitations in our study. The sample size of each group is relatively small, so large sample size is still needed to verify its reliability. There is a certain mismatch between the virtual design of percutaneous guide plate and the actual printed model, and the design software still needs to be further upgraded and optimized. Because lotus root type controller combined with percutaneous guide, and percutaneous guide contact with the skin, so in actual operation, a little shift of percutaneous guide will cause deviation of the lotus root type controller and produce puncture error, which we will explore in the later work in the case of wrong percutaneous plate deformation effectively fixed method. When we conducted the research of this project, the charge of 3D printing guide templates was still not approved in China. In the research, the guide templates were free for patients, and all the costs were covered by the research funds. The cost of each case was estimated to be 200–300 RMB.

In conclusion, the clinical effect of percutaneous guide plate combined with pointed lotus-style regulator is satisfactory. Percutaneous navigation guide punch through the skin for the first-time orientation, while pointed lotus-style regulator reach into the bone surface, avoiding the skin, muscles and other soft tissue to locate the accurate puncture, puncture in effectively reducing the number of times, operation time, perspective and accepted by the patient during radiation, reduce the postoperative patients with lower back pain symptoms, short-term improve the whole efficiency and safety operation.

**Figure 8:**
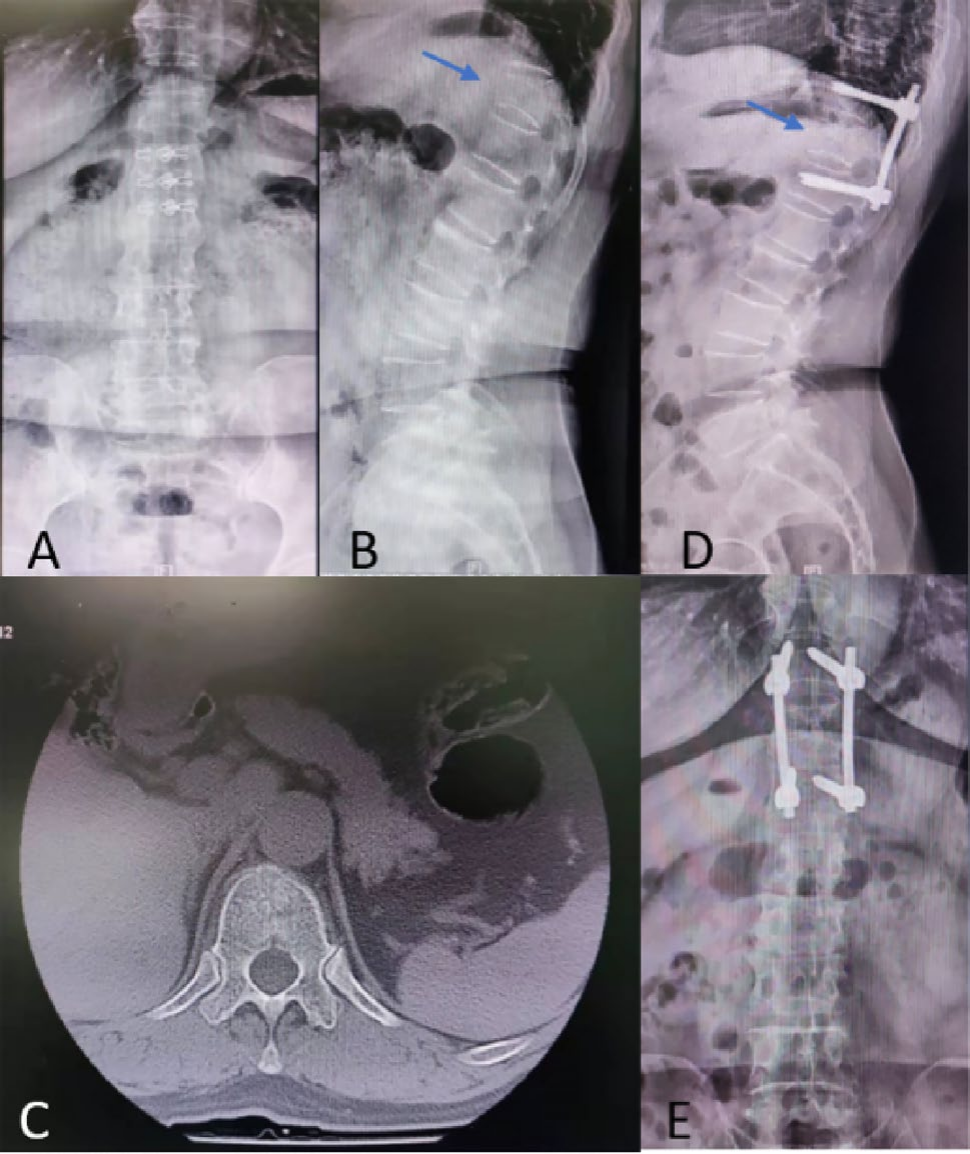
A case of AO A1.3 fracture patient. Preoperative X ray (**A**, **B**) and CT scan (**C**) and postoperative X ray (**D**, **E**).

One case report, in group B, patient Chaomei Pan, female, 63 years old, medical record No.: 1824841, was hospitalized at 15:30 a.m. on June 6, 2018 due to “low back pain caused by falling and limited activity for 1 day”. After admission, spine X-ray and CT showed 11 vertebral compression fractures, AO classification of A1.3 fracture. PPSF surgery were performed and patient were discharged with a good outcome (Fig. 8).
